# Circulating miRNAs as novel diagnostic biomarkers in hepatocellular carcinoma detection: a meta-analysis based on 24 articles

**DOI:** 10.18632/oncotarget.18949

**Published:** 2017-07-04

**Authors:** Yan Ding, Jia-Lai Yan, An-Ning Fang, Wei-Feng Zhou, Ling Huang

**Affiliations:** ^1^ Clinical Laboratory, The Second Affiliated Hospital of Southeast University, Nanjing, Jiangsu, China; ^2^ Department of Medical Technology, Anhui Medical College, Hefei, Anhui, China; ^3^ Department of Basic Medicine, Anhui Medical College, Hefei, Anhui, China; ^4^ Department of Clinical Medicine, Anhui Medical College, Hefei, Anhui, China

**Keywords:** circulating miRNAs, hepatocellular carcinoma, diagnosis, ROC, meta-analysis

## Abstract

The diagnostic value and suitability of circulating miRNAs for the detection of hepatocellular carcinoma have been inconsistent in the literature. A meta-analysis is used to systematically evaluate the diagnostic value of circulating miRNAs. Eligible studies were selected and the heterogeneity was assessed by subgroup analysis, meta-regression, and publication bias. After strictly and comprehensive screening, the source methods, internal reference and the cut-off values of the included miRNAs were first listed. Circulating miRNAs demonstrated a relatively good diagnostic value in hepatocellular carcinoma, In the subgroup analysis, diagnosis odds ratio showed a higher accuracy with multiple miRNAs than with a single miRNA as well as with serum types than plasma types. In addition, although miRNAs have many expression patterns, the high frequency expression miRNAs (miR-21, miR-199 and miR-122) might be more specific for the diagnosis of hepatocellular carcinoma.The sources of heterogeneity might be related to the number of miRNAs and the specimen types in meta-regression. Furthermore, it’s surprised that the pooled studies were first demonstrated publication bias (*P* < 0.05). In conclusion, multiple miRNAs in serum have a better diagnostic value, and the publication bias was stable. To validate the potential applicability of miRNAs in the diagnosis of hepatocellular carcinoma, more rigorous studies are needed to confirm these conclusions.

## INTRODUCTION

Hepatic celluler cancer (HCC) is the most common type worldwide, with an incidence of 780,000 new cases per year, making it the sixth malignant tumour type, while 390,000 new cases and 380,000 deaths occur in China alone [1]. Hepatitis B virus (HBV) and hepatitis C virus (HCV) cause approximately 70%–80% of all cases of cirrhosis in high-incidence areas of China [2], but HCV infection, alcohol abuse, and obesity, among other factors, may also be responsible for HCC in developed nations. Currently, due to a lack of standard symptoms and specific biomarkers, the early diagnosis of liver cancer is difficult, and the patients thus lose the chance for early surgical treatment. In the clinic, the diagnosis of HCC is usually based on the results of imaging techniques. Gadolinium -ethoxibenzyl-diethylenetriamine pentaacetic acid (Gd-EOB-DTPA) enhanced magnetic resonance imaging (MRI), abbreviated as EOB-MRI, which is the only imaging modality that has sufficient resolution for the detection and classification of early HCC. These techniques can also only detect liver cancers greater than 1 cm [3], and therefore, the diagnosis relies on pathological tests, but the most commonly used tumor marker is alpha-fetoprotein (AFP), and when 20 ng/mL is chosen as the AFP threshold, the sensitivity and specificity are 41%–65% and 80%–90% [4], respectively. Some patients, even at death, are negative for AFP because of non-secretion of AFP in the peripheral circulation [5, 6]. Prothrombin induced by vitamin K absence-I (PIVKA-II) is synthesized by the HCC-affected liver, and related to tumour size, tumour number, invasion, and metastasis [7], which sensitivity and specificity of 75.7% and 60.1%, respectively when AFP, alpha-fetoprotein variant (AFP-L3) and PIVKA-II were used jointly in the diagnosis [8]. Therefore, biomarkers with a higher degree of sensitivity and specificity are urgently needed for diagnostic and prognostic indicators in the clinic.

MiRNAs are small non-coding single-stranded RNAs (18 to 24 nucleotides long) that interact with their target mRNAs to inhibit translation via the promotion of mRNAs degradation or to block translation by binding to complementary sequences in the 3′- untranslated region of mRNAs. MiRNAs are involved in various biological processes, including proliferation, differentiation, signal transduction, fat metabolism and apoptosis, which affect the growth and development at the cellular, tissue or organism level; they also play a role in the occurrence and development of a variety of diseases [9, 10]. Furthermore, miRNAs are well protected from RNase and remain stable during exposure to harsh conditions [11]. Mounting evidence has shown that miRNAs can be used as potential biomarkers in the diagnosis of various types of human cancer, as follows: digestive tract cancers, breast cancers [12, 13], and HCC [14, 15]. Although miRNAs are specific to liver cancer tissues and covered by an envelope, which means that they are not easily secreted into the blood, but experiments have also shown that circulating miRNAs may be detected in human serum or plasma because the miRNAs of membranous vesicles are secreted and then bind to RNA-related proteins or high-density lipoprotein cholesterol [16, 17]; these can be released by damaged or compromised cells. Many similar theories have been described and gaven the specific miRNA and specific target genes in HCC, for example low-expression of miRNA-139 in HCC may suppress metastasis and progression of cancer cells by down-regulating Rho-kinase 2 [18].

Based on those theories research, a series studies have suggested that it is possible to use serum/plasma miRNAs as novel non-invasive biomarkers to diagnosed HCC [19–25]. However, no definite consensus has emerged on the miRNAs that are specifically associated with HCC. This may be in part attributed to the presence of different miRNA-related subclasses of HCC, different sample style, different control, different cutoff value and so on. Li et al. [19] firstly studied the miRNA expression spectrum in 2010, they used miRNAs (miR-10 and miR-125b) for the detection of HBV-positive HCC from HBV group with 98.5% sensitivity and 98.5% specificity, but when Ghosh et al. [25] showed a miR-126, sensitivity and the specificity were relatively lower: only 63% and 58%, respectively. Although it is not easy to use miR-122 to distinguish chronic hepatitis B from liver cancer [21] and despite its lower diagnostic value compared with that of AFP [22], this miRNA can be used as a prognostic indicator because the serum miR-122 level is positively related to the survival rate of patients with liver cancer [23]. But others have found a negative correlation between elevated serum miR-122 levels and the prognosis of liver cancer [24]. In addition, snRNAU6 was used as qRT- PCR control in some articles, while miR-16 or other miRNAs was used in others. Therefore, there have been inconsistencies and even contradictions in the literature regarding the reliability of circulating miRNAs for the early detection of HCC [26–28]. In this meta-analysis, an overview of circulating miRNAs present in peripheral blood is given to further clarify the clinical value of miRNAs in terms of HCC diagnosis and to provide more comprehensive reference information for the early detection of HCC.

## MATERIALS AND METHODS

### Literature selection

This meta-analysis was conducted according to the guidelines for the diagnostic meta-analytic method. We searched PubMed, Embase, the Cochrane Library and Web of Science, without language limitation, and the final article retrieval was completed before March 20, 2016. The following retrieval strategy was used: (‘liver tumors’ OR ‘hepatocellular carcinoma’ OR ‘primary liver cancer’ OR ‘liver cancer’) AND (‘tiny RNA’ OR ‘microRNA’ OR ‘major miRNAs’ OR ‘circular small RNA’ OR ‘cycle of miRNAs’) AND (‘diagnosis’ OR ‘sensitivity and specificity’ or ‘the receiver work features’ OR ‘ROC curve’ OR ‘forecast’). In addition, the reference lists of eligible articles were independently and manually searched to obtain supplementary studies.

### Literature inclusion and exclusion criteria

Studies that were included in our meta-analysis met the following criteria: (1) Studies had the full text available regarding the use of circulating miRNAs in peripheral blood for HCC diagnosis; (2) all cases and controls were not restricted by age or race, and all were clinically diagnosed by the gold standard; and (3) access was provided to the original measurement data, including the first author, year and country, case group and control group, sample size, age, type of specimens, type of miRNA assay, methods of miRNAs detection as well as the threshold, sensitivity, specificity and information needed for the quality assessment and QUADAS score. The exclusion standards were as follows: (1) the reports were meeting reports, reviews, or comments; (2) the pathological diagnosis standard was obscure; (3) full data could not be obtained; and (4) the studies used repeated data or and data of poor quality.

### Quality assessment

The quality of the included studies was scored independently by two reviewers according to The Revised Tool for the Quality Assessment of Diagnostic Accuracy Studies-2 (QUADAS-2) criteria [29]. Four key domains comprise these guidelines as follows: patient selection, index test, reference standard and flow and timing. Each domain contains seven questions, which can be answered by “yes” “no” or “not clear,” that assess the quality of included studies. An answer of “yes” means a low risk of bias, whereas “no” or “not clear” means a higher risk of bias in terms of the loss of some information from the literature. In cases of conflict, a third reviewer was consulted, controversies were settled through discussion, and the results were finally decided. Three aspects were used to determine the applicability of the articles: reference, case selection, and inspection. The applicability of the entire high risk of bias is lower than for the indicators.

### Statistical analysis

Using the Stata 12.0 software to perform the meta-analysis, the Spearman test was used to analyse the threshold effect or the non-threshold effect. The between-study heterogeneity was evaluated by *Q* test and *I*^2^ statistic. If the *P* value was less than 0.10 or if the *I*^2^ value was more than 50%, the random effects model was selected. Furthermore, a subgroup analysis and regression analysis were performed to explore the potential sources of heterogeneity [30, 31]. We summarized the pooled sensitivity (SEN), the pooled specificity (SPE), the positive likelihood ratio (PLR), the negative likelihood ratio (NLR) and the diagnostic odds ratio (DOR) [32]. In addition, the summary receiver-operating characteristics (SROC) curve was generated and the AUC was calculated both overall and for the subgroup analysis. Furthermore, a funnel plot was used to detect publication bias, and *P* < 0.10 indicated definite publication bias [33]. All of the statistical tests were two-sided, and *P* < 0.05 was considered statistically significant.

## RESULTS

### Basic information of the included studies

**Figure 1:**
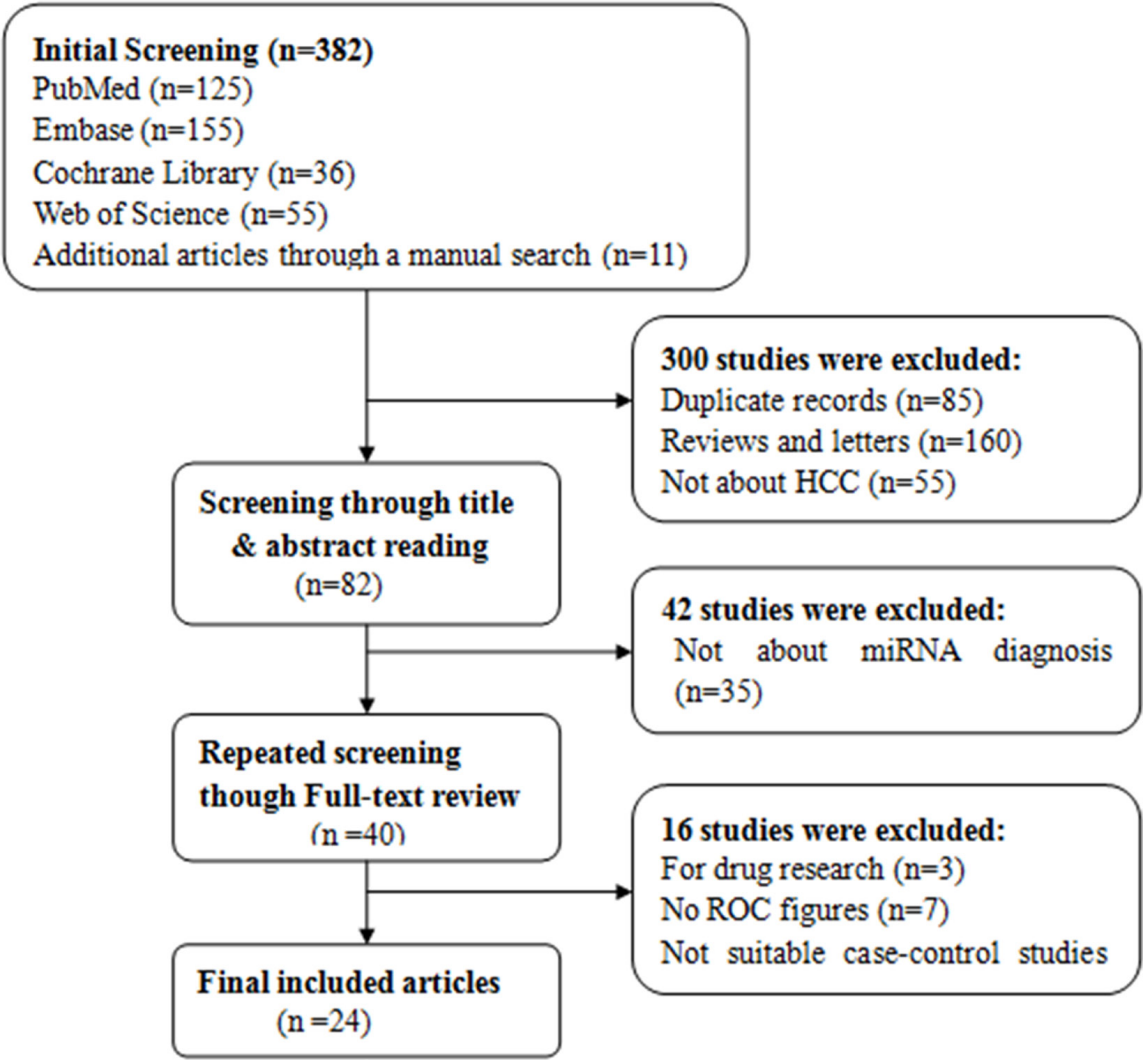
Studies evaluated with criteria for exclusion and inclusion

**Figure 2:**
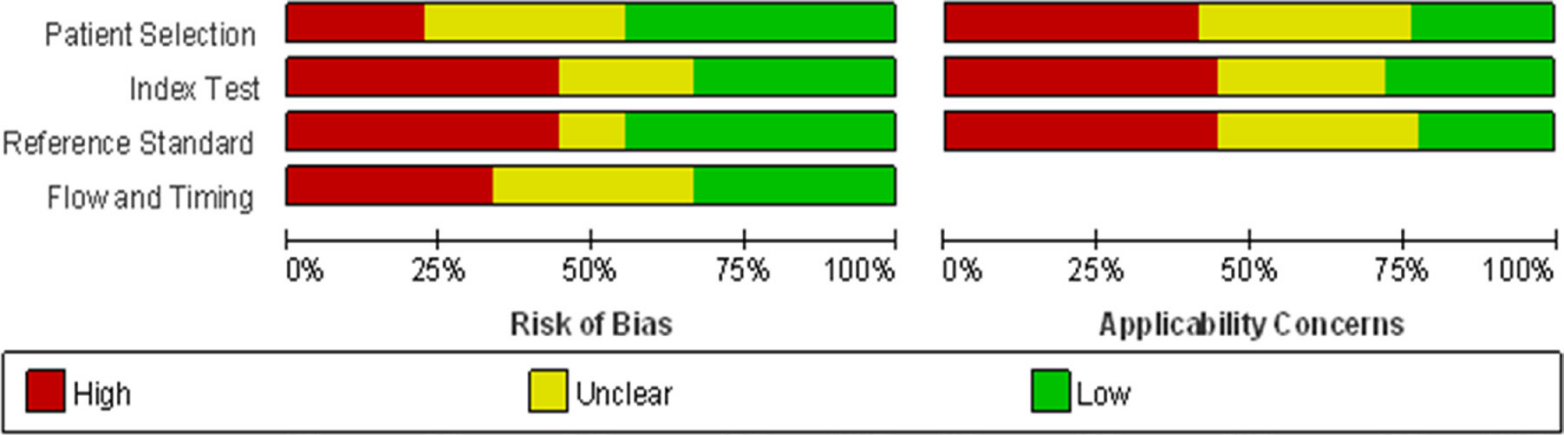
Overall quality assessment of included articles using the QUADAS-2 criteria (a: proportion of articles with high, mediate or low risk of bias; b: proportion of articles with high, mediate or low concerns regarding applicability)

According to the literature retrieval strategy, the initial search returned a total of 382 articles, of which 300 articles were duplicates, reviews, news reports, meeting records and others that did not focus on HCC and were thus excluded. Then, 47 articles remained for title and abstract review, which resulted in 40 articles available for full text review. After further careful review, 16 were removed for lack of sufficient information. Eventually, 24 [3, 5, 19, 21, 25, 26, 34–51] articles were included in our meta-analysis, and all were published in English. In all, 58 studies from 24 articles, including 2193 patients with hepatocellular carcinoma and 2347 people as control population. 1531, 816 controls were patients with hepatitis B/C or cirrhosis and healthy individuals, respectively (Figure 1). The characteristics of the 24 articles are listed (Supplementary Table 1). The publication years and the number of articles were 2016 (*n* - 3), 2015 (*n* - 7), 2014 (*n* - 3), 2013 (*n* - 2), 2012 (*n* - 4), 2011 (*n* - 4) and 2010 (*n* - 1). 18 articles contained studies that were conducted in China, whereas 6 articles in other nations. Although all of the studies used quantitative real-time reverse transcription-PCR (qRT-PCR) to measure the expression of miRNAs, some performed this method directly, whereas others first conducted screening microarray, TaqMan low density array (TLDA) chips and high-throughput sequencing.Some studies used snRNAU6 as an internal control, whereas others used miR-16, miR-39 and so on. Moreover, the cut-off values of different miRNAs for diagnosis HCC varied from –3.24 to 19.18. Of 58 included studies, 32 investigated the differential expression of miRNAs between HCC patients and healthy controls, whereas the patient groups in 39 studies were composed of more patients with HBV-related chronic hepatitis or cirrhosis and fewer patients with HCV-related hepatitis or non-virus infection patients. In all, 43 miRNA studies focused on a single miRNA, and 15 studies focused on multiple miRNAs. Furthermore, the high frequency expression miRNAs (miR-21,miR-199 and miR-122) may be more specific for the diagnosis of primary liver cancer. 39 studies used serum specimens and 19 plasma samples for qRT-PCR. Quality assessments are shown in a bar graph with QUADAS-2 scores in Figure 2; the figure indicates that the quality of the research was moderate-high with scores greater than 4.

### Threshold effect

By drawing the ROC curve and calculating the Spearman correlation coefficient using the logarithm of sensitivity and the logarithm of (1 - specificity), the threshold effect was assessed. The results showed that the shape of the ROC curve not like shoulder and arm shaped distributed. The Spearman correlation coefficient was –0.085 and the *P* value was 0.535 (*P* > 0. 05), which indicates no threshold effect.

### Diagnostic accuracy of circulating miRNAs in the peripheral blood of HCC patients

**Figure 3:**
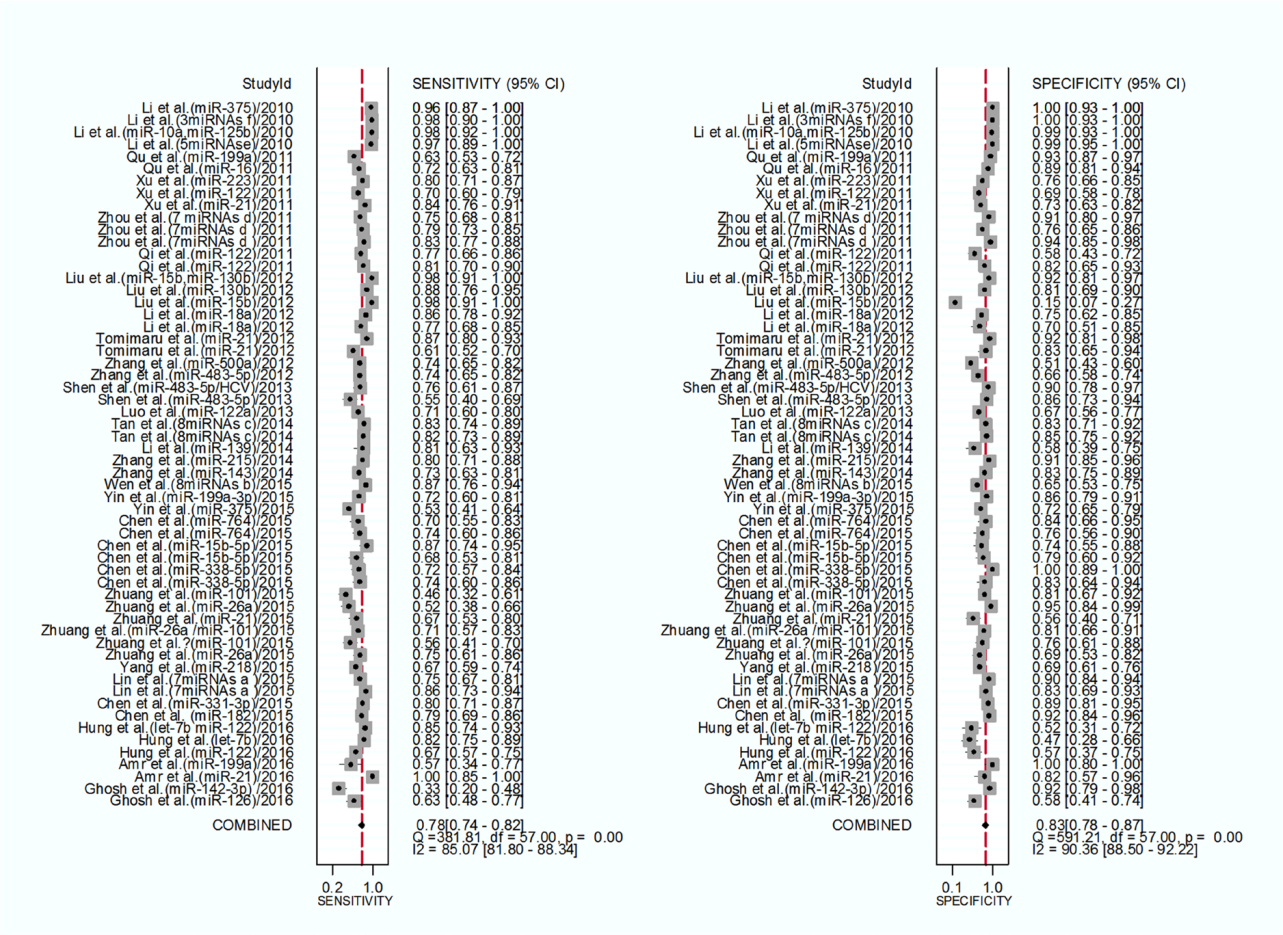
Forest plot of the sensitivities and specificities of circulating miRNAs for the diagnosis of hepatocellular carcinoma The point estimates the sensitivity and specificity among the studies as solid squares. Error bars with 95% confidence intervals (CIs).

**Figure 4:**
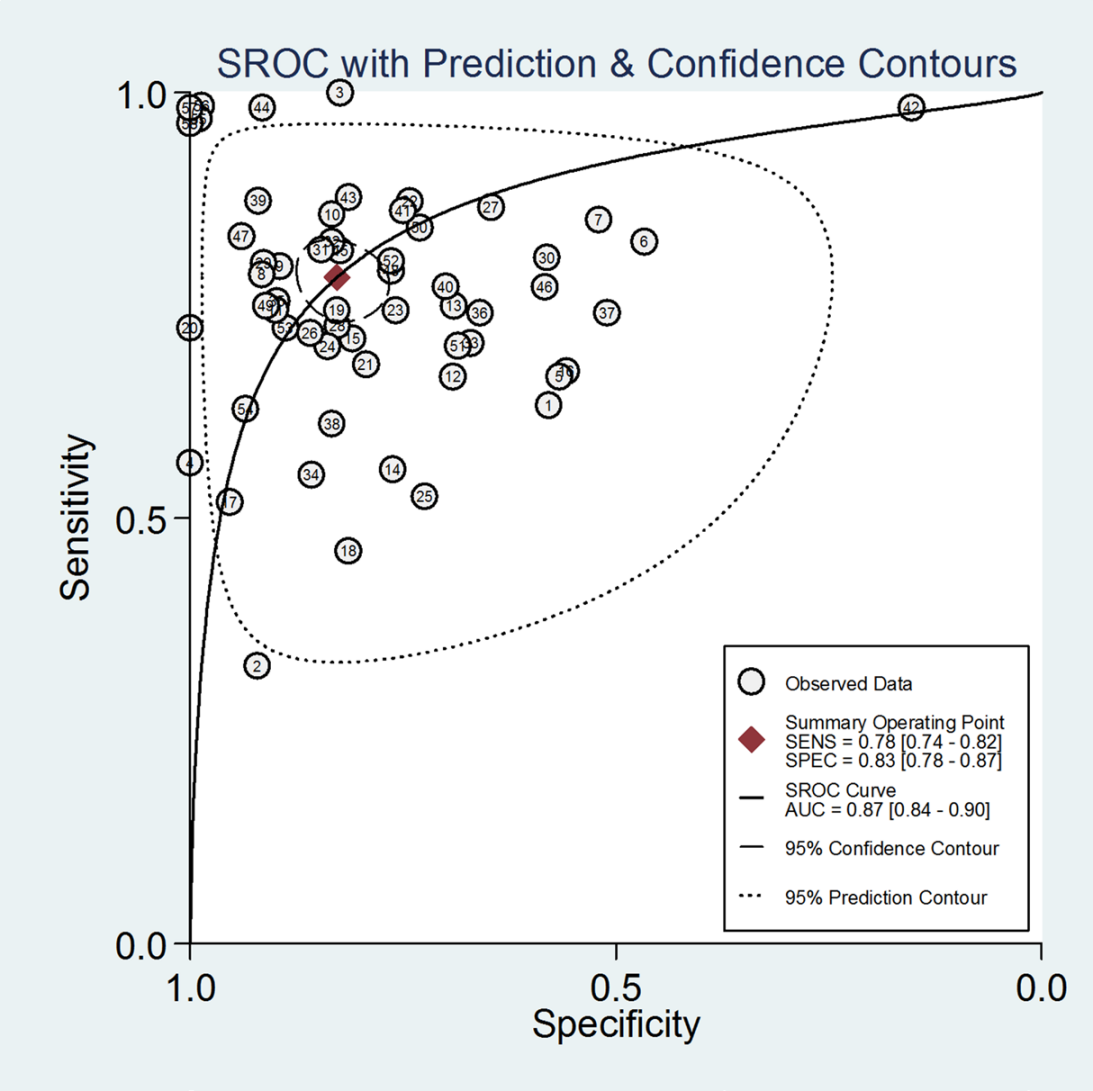
Summary receiver operating characteristic (SROC) curve with pooled estimates of sensitivity, specificity and AUC on the diagnostic value of circulating miRNAs in HCC The number in each circle corresponds to the number of studies.

Forest plots were used to analyse the sensitivities and specificities of the 58 miRNAs in the peripheral blood circulation of HCC patients in the diagnosis of HCC. From the sensitivity and specificity data (*I*^2^ = 85.07%, *I*^2^ = 90.36%, respectively) (Figure 3), significant heterogeneity among studies was observed and the random effects model for the meta-analysis was adopted. The pooled parameters were calculated as follows: sensitivity, 0.78 (95% CI: 0.74∼0.82), specificity, 0.83 (95% CI: 0.78∼0.87), PLR, 4.5 (95% CI: 3.5∼5.9), NLR, 0.26 (95% CI: 0.22∼0.32), DOR, 17.0 (95% CI: 12∼26) and AUC, 0.87 (95% CI: 0.84∼0.90). (Figure 4) (Table 1).The results showed that miRNAs had a relatively high diagnostic accuracy.

**Table 1:** Summary estimates of diagnostic criteria and their 95% confidence intervals

Subgroups		Sensitivity (95% CI)	Specificity (95% CI)	Positive LR (95% CI)	Negative LR (95% CI)	DOR (95% CI)
Internal reference types in qRT-PCR						
U6	23	0.71 [0.66-0.75]	0.80 [0.74-0.85]	3.5 [2.7–4.6]	0.36 [0.31–0.42]	10 [7–14]
Non-U6	22	0.76 [0.71–0.81]	0.80 [0.74–0.86]	3.9 [2.9–5.3]	0.30 [0.24–0.37]	13 [9–20]
Source of control						
Healthy control	19	0.77 [0.71–0.82]	0.85 [0.79–0.90]	5.2 [3.5–7.6]	0.27 [0.20–0.36]	19 [10–36]
Chronic liver disease	39	0.79 [0.74–0.84]	0.81 [0.75–0.86]	4.2 [3.1–5.8]	0.26 [0.20–0.33]	16 [10–27]
Regulation mode						
Up-regulation	45	0.82 [0.77–0.85]	0.83 [0.77–0.88]	4.8 [3.5–6.6]	0.22 [0.18–0.28]	22 [13–36]
Down-regulation	13	0.65 [0.59–0.70]	0.82 [0.74–0.88]	3.5 [2.5–5.0]	0.43 [0.37–0.50]	8 [5–13]
MiRNA profiling						
Single miRNA	43	0.74 [0.70–0.78]	0.79 [0.74–0.84]	3.6 [2.8–4.6]	0.32 [0.27–0.38]	11 [8–16]
Multiple miRNAs	15	0.87 [0.80–0.92]	0.90 [0.82–0.95]	8.8 [4.6–16.9]	0.14 [0.09–0.23]	62 [21–180]
Specimen types						
Plasma	19	0.74 [ 0.68–0.79]	0.81 [0.74–0.87]	4.0 [2.8 -5.6]	0.32 [0.26–0.40]	12 [8- 20]
Serum	39	0.81 [0.75–0.85]	0.83 [0.77- 0.88]	4.9 [3.4–6.9]	0.23 [0.18- 0.30]	21 [12–37]
Cut-off						
< 1.00	13	0.79 [0.72–0.85]	0.75 [0.67–0.83]	3.2 [2.2–4.6]	0.28 [0.19–0.40]	12 [6–23]
> 1.00	28	0.71 [0.67–0.76]	0.81 [0.75–0.85]	3.7 [2.8–4.7]	0.36 [0.30–0.42]	10 [7–15]
Country						
Chinese	45	0.80 [0.76–0.83]	0.83 [0.77–0.87]	4.6 [3.4–6.3]	0.24 [0.19–0.30]	19 [12–31]
Non-chinese	13	0.71 [0.61–0.80]	0.83 [0.72–0.90]	4.2 [2.6–6.8]	0.35 [0.25–0.47]	12 [6–23]
Total	58	0.78 [0.74–0.82]	0.83 [0.78–0.87]	4.5 [3.5–5.9]	0.26 [0.22–0.32]	17 [12–26]
(Excluded 6 studies)Total	52	0.75 [0.71–0.78]	0.80 [0.75–0.83]	3.7 [3.0–4.4]	0.32 [0.28–0.36]	12 [9–15]

*LR*: likelihood ratio, *DOR*: diagnostic odds ratio, *AUC*: area under the curve, *CI*: confidence interval.

### Subgroup analysis and regression analysis

A subgroup analysis was conducted according to qRT-PCR internal references, source of controls, mode of miRNA regulation, miRNAs profiling, specimen types miRNA, cut-off values and countries. The pooled sensitivity, specificity, PLR, NLR and DOR for each subgroup analysis were listed (Table 1), and we found that Multiple miRNA assays had a better diagnostic value than single miRNA assays: sensitivity (0.87 vs. 0.74), specificity (0.90 vs. 0.79), PLR (8.8 vs. 3.6), NLR (0.14 vs. 0.32), are DOR (62.0 vs. 11.0). Serum types had also a better diagnostic value than plasma types: sensitivity (0.81 vs. 0.74), specificity (0.83 vs. 0.81), PLR (4.9 vs. 4.0), NLR (0.23 vs. 0.32), are DOR (21.0 vs. 12.0). Apart from that the up-regulated miRNAs were slightly better than the down-regulated miRNAs in the diagnosis of HCC: detection sensitivity (0.82 vs. 0.65), specificity (0.83 vs. 0.82), PLR (4.8 vs. 3.5), NLR (0.22 vs. 0.43), and DOR (22.0 vs. 8.0). Using healthy individuals as controls was also slightly efficient diagnostic value than using chronic liver disease. Internal references, cut-off values and countries had no impact on the diagnosis (Table 1).

The odds ratio (OR) is used in the meta-regression analysis for the binary classification of variable data. LogOR was used as the response variable. Source of controls, miRNA profiling, regulation modes, countries and specimen types were as covariates. The results showed that this regression method had statistically significant *P* = 0.05. the I-squared-res value was 33.61%, and thus the heterogeneity could be explained by 33.61% of the residual variation (another 66.39% of residual variation was explained among the studies). The adjusted R-squared was 36.81%, according to the covariate model, which could explain the variation among the studies; this variation, which was 36.81%, might be related to the miRNA profiling (*P* = 0.06) and specimen types (*P* = 0.07) (Table 2), but was not related to source of controls, regulation modes and country types. The internal references and cut-off values didn’t conclude in this regression because of missing some data and no universal standard (Supplementary Table 1).

**Table 2:** Using the odds ratio (OR) for the meta-regression analysis in the binary classification of variable data

LogOR	exp(b)	Std. Err	*t*	*P*>|t|	[95% Conf.Interval]	
Source of control	1.119288	0.1970724	0.64	0.525	0.7861435	1.59361
MiRNA profiling	0.6951539	0.1334742	–1.89	0.064	0.472881	1.021904
Regulation modes	1.202073	0.2335982	0.95	0.348	0.8139118	1.775351
Country types	1.247222	0.2363241	1.17	0.249	0.8527394	1.824195
Specimen types	0.7257147	0.1261222	–1.84	0.071	0.5120494	1.028537

LogOR was used as response variables as well as source of controls, miRNA profiling, regulation modes, countries and specimen types were as covariates Estimate ofbetween-study variance tau2 = 0.03685. Residual variation due to heterogeneity: I-squared_res = 33.61%. Proportion of between-study variance explained: Adj R-squared = 36.81%. Joint test for all covariates with Knapp-Hartung modification: Prob > F = 0.0509.

### Sensitivity analysis

For the abnormal value analysis, we chose the sensitivity for the variable Z, and selected a new variable value of more than 2 for outliers in the statistical description in SPSS 17.0. Articles excluded [19, 26, 49] because of the sensitivity was too high. A comparison of the new results with the original overall results showed the following: the sensitivity was 0.75 vs. 0.78, the specificity was 0.80 vs. 0.83, the PLR was 3.7 vs.4.5, the NLR was 0.32 vs.0.26, the DOR was 12.0 vs.17.0 and the AUC was 0.83 vs. 0.87 (Table 1). This change showed no obvious influence and indicates that the previous pooled result was stable.

### Publication bias

**Figure 5:**
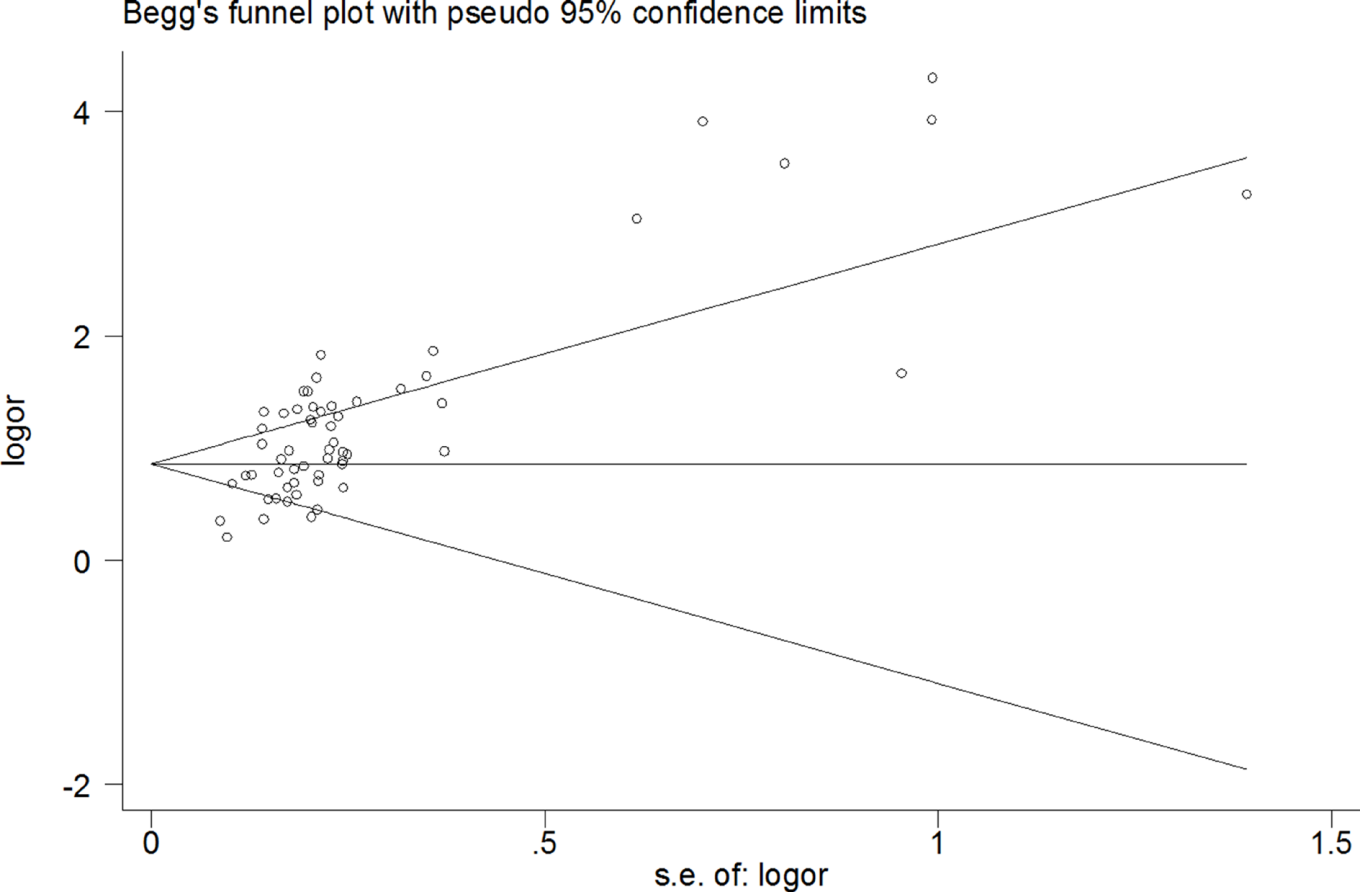
The pooled Egger’s test of the diagnostic meta-analysis (*t* = 6.94, *P* < 0.00)

**Figure 6:**
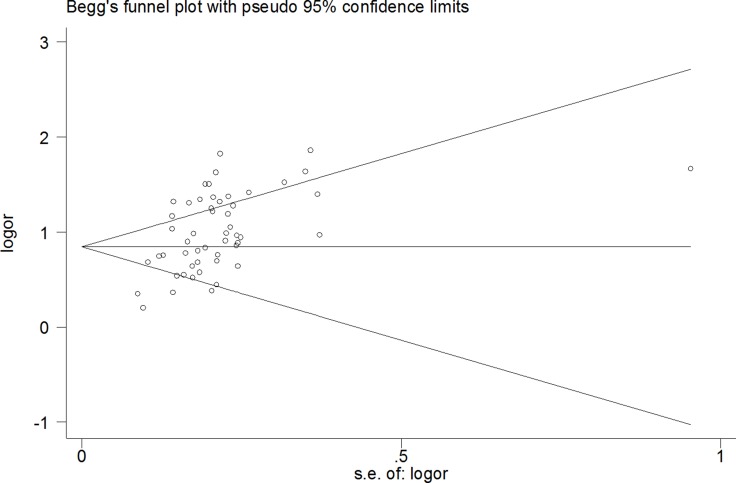
The pooled Egger’s test of the diagnostic meta-analysis with deleted six outline data (*t* = 5.14, *P* < 0.00)

A funnel diagram tested potential publication bias. The pooled Egger’s test results were *t* = 6.94, *P* < 0.00, 95% CI: 2.84∼5.14 (Figure 5), The results were still same *t* = 5.14, *P* < 0.00 excluded [19, 26, 49], there was publication bias (Figure 6). In addition, when healthy individuals were used as controls, the Egger’s test results were *t* = 2.95, *P* < 0.009, but when the one result of Li et al.(5 miRNAse) was deleted, there was no publication bias (*t* = 1.75, *P* > 0.10). However, when patients with liver disease were used as controls, the results were *t* = 5.75, *P* < 0.000 (95% *CI*: 2.37∼4.95), which also indicated statistically significant publication bias with excluded another five studies.

## DISCUSSION

Liver cancer has a great propensity for invasion and metastasis and usually has a poor prognosis [52]. Because the integration of the genome of the HBV X gene in liver cells results in the suppression of P53 protein and the activation of proto-oncogenes [53]. The diagnosis of liver cancer is very limited because terminally ill patients only have less than a 20% chance of survival for longer than one year after the initial diagnosis [54]. Therefore, a more sensitive and specific non-invasive biomarker is urgently needed for the diagnosis of early HCC. The potential reasons of miRNA in the diagnosis of HCC are superiorities as compared with current markers [55], such as AFP, may be that the former more sensitive using PCR method and many various miRNA profiling, which could be combined each other to improve diagnostic accuracy. Alteration of expression levels of miRNA are biological significance, such as carninogenesis and cell proliferation. MiRNA-182 [56] contributes to metastasis of HCC by down-regulation metastasis suppressor 1 and increases drug resistance in cisplatin-treated HCC cell by regulating tumor protein 53-induced nuclear protein 1. MiR-331–3P [57] promotes proliferation and metastasis of HCC by targeting PH domain and leucine-rich repeat protein phosphatise. MiRNA-21 is upregulated in HCC cells and tissues, which are associated with the capacity of cancer cell migration and invasion in HCC [58]. Plasma miRNA-21 levers are significantly reduced in the post-operative [3], The expression of miRNA-122 is down regulated in HCC tumor tissues and cancer cell lines and overexpression of miRNA-122 has been found to induce apoptosis and suppress proliferation in HepG2 and Hep3B cells [59]. MiRNA-199a-3p levels were inversely correlated with mammalian target of rapamycin protein expression in human HCC samples [60]. The above studies demonstrated that circulating miRNAs could be non-invasive diagnostic or prognostic markers for HCC. This meta-analysis on the diagnostic values of miRNAs for HCC was done to system and detail analysis.

Hu [6] first conducted a meta-analysis on the diagnostic values of miRNAs from eight pooled articles. Yin [61] analysed the hierarchical subgroups among 14 studies according to the different controls used (healthy controls and patients with chronic liver disease) and miRNA profiling (single miRNAs and multiple miRNAs). He [62] analyzed ROC that combination of AFP and miRNAs, and also calculated diagnostic accuracy though comparing every subgroup. They showed best three panels for HCC diagnosis were studied by Li et al. [19] such as the panel of miR-10a and miR-125b with sensitivity of 0.98, specificity of 0.98,and AUC of 0.99. They also showed single miRNAs with highest AUC-SROC among their included studies. In our meta-analysis, the high frequency expression miRNAs (miR-21, miR-199 and miR-122) may be more specific for the diagnosis of HCC, which emerged in different articles. This studies were screened more strictly. For example, the up-regulation of miR-650 and the down-regulation of miR-618 in the urine of HCV-positive hepatitis patients could aid in the diagnosis of HCC for a follow-up of two years [28]. Combined diagnoses using miRNAs and AFP [25], rather than solely a combination of miRNAs, have been discounted. The serum miR-486-5p level is likely able to be used for the diagnosis of liver cancer recurrence after surgery [63]. We first considered that the source of the heterogeneity may be related to the miRNAs regulation mode, internal references and the cut-off values of the included miRNAs in qRT-PCR strategy.These comprehensive messages were listed for the diagnostic value of a particular miRNA. Which internal reference used to check miRNAs in RT-PCR are disputed. Chen [43] mentioned that snRNAU6 could not be detected in almost half of plasma samples while miR-16 was abundant in all plasma samples. Tomimaru [3] also mentioned miR-16 were rich in plasma while RNU48 in tissue. In our studies, it was easily found that U6 was firstly used more common, miR-16, miR-39 came secondly. It is well known that cut-off values affect the diagnosis. The cut-off values of the included miRNAs varied from –3.24 to 19.18. It was hard to say universal standard.

In this study, the NLR showed that the diagnosis of HCC using miRNAs had a 26% false negative rate, and thus, the use of circulating miRNAs in the detection of HCC has some deficiencies. The pooled DOR signified that the chance of a correct diagnosis of HCC was 17 times greater than a false-negative diagnosis of non-HCC patients. Large heterogeneity was found among the included studies, although some believe that a meta-analysis is meaningless unless it is merged with some overall statistical results. Others believe that the existence of heterogeneity may be due to the time of study publication, the research group, or the study of the characteristics of objects and other factors. As long as a subgroup analysis or meta-regression analysis can be used to control or interpret heterogeneity, the bias caused by various factors may be eliminated. In our study, the subgroup analysis or meta-regression analysis showed that multiple miRNAs and serum type had better diagnostic value. It has been widely accepted that miRNAs in plasma are rich than in serum sample [55]. Perhaps most articles selected serum as samples lead to the serum type had better diagnostic value [64]. The heterogeneity of the control types and regulation types had little influenced. A possible for this reason, most hepatitis-disease control groups contained no symptom hepatitis carriers, which caused nearly the similar results compared to the healthy donors groups. This result was accordance with He et al [62]. The funnel diagram tested potential publication bias, which firstly indicates publication bias, but not agree with other reports in the literature [6, 62]. Perhaps with more studies in miRNA_S_, more likely to report positive results. In addition, heterogeneity may be related to different methods of screening miRNAs, no unified primers, internal miRNAs or cut-off values. Due to the lack of some data in the included studies, we were not able to analyse the potential relationship between the level of miRNAs and the clinical-pathological characteristics of HCC. The source of miRNA expression also varies (e.g. liver tissue, peripheral blood).

This meta-analysis may have some deficiencies: (1) Some related references may have been omitted, or portions of the data may have been omitted from the study; (2) Different miRNAs and cut-off values may lead to contradictory results; (3) Publication bias is evident in the funnel figure of the pooled data; and (4)This meta-analysis did not consider the internal references and cut-off values in meta-regression analysis due to a lack of sufficient data. Despite these shortcomings, our meta-analysis indicated that although miRNAs have many expression patterns, the high frequency expression miRNAs (miR-21, miR-199 and miR-122) may be more specific for the diagnosis of HCC, although they also reflect other liver injury or other tumors. Multiple miRNAs in serum have a better diagnostic value, To validate the potential applicability of miRNAs in the diagnosis of HCC, more rigorous studies are needed to confirm these conclusions.

## SUPPLEMENTARY MATERIALS TABLE





## Abbreviations

HCC: Hepatocellular carcinoma
DOR: Diagnosis odds ratio
Gd-EOB-DTPA: Gadolinium -ethoxibenzyl-diethylenetriamine pentaacetic acid
MRI: Magnetic resonance imaging
PIVKA-II: Prothrombin induced by vitamin K absence-II
miRNAs: MicroRNAs
SEN: Sensitivity
SPE: Specificity
PLR: Positive likelihood ratio
NLR: Negative likelihood ratio
SROC: Summary receiver-operating characteristics
qRT-PCR: Quantitative real-time reverse transcription-PCR
OR: Odds ratio
TLDA: TaqMan low density Array
Cons: Constant
