# Supplementary material for: Circulating miRNAs as novel diagnostic biomarkers in hepatocellular carcinoma detection: a meta-analysis based on 24 articles
Source: Oncotarget. 2017 Jul 4;8(39):66402–13. doi: 10.18632/oncotarget.18949 (PMC5630422; doi:10.18632/oncotarget.18949)
Supplement: Supplementary file 1 [file oncotarget-08-66402-s001.pdf]

Circulating miRNAs as novel diagnostic biomarkers in hepatocellular carcinoma detection: a meta-analysis based on 24 articles

Supplementary Materials

Supplementary Table 1: Main characteristics of the 24 articles on hepatocellular carcinoma included in the meta-analysis. See Supplementary_Table_1
