# Supplementary material for: Circulating miRNAs as novel diagnostic biomarkers in hepatocellular carcinoma detection: a meta-analysis based on 24 articles
Source: Oncotarget. 2017 Jul 4;8(39):66402–13. doi: 10.18632/oncotarget.18949 (PMC5630422; doi:10.18632/oncotarget.18949)
Supplement: Supplementary file 2 [file oncotarget-08-66402-s002.docx]

**Supplementary Table 1: Main characteristics of the 24 articles on hepatocellular carcinoma included in the meta-analysis**

| **First Author** | **Year** | **Country** | **Specimen** | **Method** | **Sample size** | | **HCC** | |
| --- | --- | --- | --- | --- | --- | --- | --- | --- |
|  |  |  |  |  | **Cases** | **Controls** | **Age±SD (years)** | **HBV/HCV** |
| Ghosh et al.[25] | 2016 | India | plasma | microarray-qRT-PCR | 49 | 38 | NR | HBV |
|  |  |  | plasma | microarray-qRT-PCR | 49 | 38 | NR | HBV |
| Amr et al.[49] | 2016 | India | serum | qRT-PCR | 23 | 17 | 54.27±9.18 | 81%HCV |
|  |  |  | serum | qRT-PCR | 23 | 17 | 54.27±9.18 | 81%HCV |
| Hung et al.[48] | 2016 | Taiwan | serum | microarray-qRT-PCR | 120 | 30 | 58.5±10 | HBV |
|  |  |  | serum | microarray-qRT-PCR | 120 | 30 | 58.5±10 | HBV |
|  |  |  | serum | microarray-qRT-PCR | 67 | 25 | 58.5±10 | HBV |
| Chen et al.[47] | 2015 | China | serum | qRT-PCR | 103 | 95 | 52 | 70%HBV |
|  |  |  | serum | qRT-PCR | 103 | 95 | 52 | 70%HBV |
| Lin et al.[46] | 2015 | China | serum | microarray -qRT-PCR | 49 | 42 | 47 | NR |
|  |  |  | serum | microarray -qRT-PCR | 153 | 139 | 49 | NR |
| Yang et al.[45] | 2015 | China | serum | qRT-PCR | 156 | 162 | 53.7 | 72% HBV |
| Zhuang et al.[44] | 2015 | China | serum | qRT-PCR | 52 | 42 | 51.1±1.4 | 63.4%HBV |
|  |  |  | serum | qRT-PCR | 52 | 42 | 51.1±1.4 | 63.4%HBV |
|  |  |  | serum | qRT-PCR | 52 | 42 | 51.1±1.4 | 63.4%HBV |
|  |  |  | serum | qRT-PCR | 52 | 43 | 51.1±1.4 | 63.4%HBV |
|  |  |  | serum | qRT-PCR | 52 | 43 | 51.1±1.4 | 63.4%HBV |
|  |  |  | serum | qRT-PCR | 52 | 43 | 51.1±1.4 | 63.4%HBV |
| Chen et al.[43] | 2015 | China | plasma | microarray-qRT-PCR | 47 | 29 | 45 | NR |
|  |  |  | plasma | microarray-qRT-PCR | 47 | 31 | 45 | NR |
|  |  |  | plasma | microarray-qRT-PCR | 47 | 29 | 45 | NR |
|  |  |  | plasma | microarray-qRT-PCR | 47 | 31 | 45 | NR |
|  |  |  | plasma | microarray-qRT-PCR | 47 | 29 | 45 | NR |
|  |  |  | plasma | microarray-qRT-PCR | 47 | 31 | 45 | NR |
| Yin et al.[42] | 2015 | China | serum | qRT-PCR | 78 | 156 | 56.3±6.7 | 62% HBV |
|  |  |  | serum | qRT-PCR | 78 | 156 | 56.3±6.7 | 62% HBV |
| Wen et al.[41] | 2015 | China | plasma | TLDA Chips-qRT-PCR | 67 | 82 | 40 | HBV |
| Zhang et al.[40] | 2014 | China | serum | qRT-PCR | 95 | 127 | 54.21±6.95 | NR |
|  |  |  | serum | qRT-PCR | 95 | 127 | 54.21±6.95 | NR |
| Li et al.[39] | 2014 | China | plasma | microarray-qRT-PCR | 31 | 31 | 49±11 | HBV |
| Tan et al.[38] | 2014 | China | serum | sequencing-qRT-PCR | 103 | 78 | 52.01±10.21 | HBV |
|  |  |  | serum | sequencing-qRT-PCR | 103 | 60 | 52.01±10.21 | HBV |
| Luo et al.[37] | 2013 | China | serum | qRT-PCR | 85 | 85 | 53.6±12.0 | HBV |
| Shen et al.[36] | 2013 | USA | plasma | TLDA Chips-qRT-PCR | 49 | 49 | 61.1±11.7 | 66%HCV |
|  |  |  | plasma | TLDA Chips-qRT-PCR | 49 | 49 | 61.1±11.7 | 66%HCV |
| Zhang et al.[35] | 2012 | China | plasma | microarray-qRT-PCR | 112 | 141 | 54.4±12.9 | HBV |
|  |  |  | plasma | microarray-qRT-PCR | 112 | 141 | 54.4±12.9 | HBV |
| Tomimaru et al.[3] | 2012 | Japan | plasma | qRT-PCR | 126 | 30 | 63±10 | 69%HCV |
|  |  |  | plasma | qRT-PCR | 126 | 50 | 62±8 | 69%HCV |
| Li et al.[34] | 2012 | China | serum | qRT-PCR | 101 | 30 | 54±11 | HBV |
|  |  |  | serum | qRT-PCR | 101 | 60 | 54±11 | HBV |

| Liu et al.[26] | 2012 | China | serum | qRT-PCR | 57 | 59 | 60 | HBV |
| --- | --- | --- | --- | --- | --- | --- | --- | --- |
|  |  |  | serum | qRT-PCR | 57 | 59 | 60 | HBV |
|  |  |  | serum | qRT-PCR | 57 | 59 | 60 | HBV |
| Qi et al.[21] | 2011 | China | serum | qRT-PCR | 70 | 34 | 49 | HBV |
|  |  |  | serum | qRT-PCR | 70 | 48 | 49 | HBV |
| Zhou et al. [5] | 2011 | China | plasma | microarray-qRT-PCR | 196 | 66 | 53±12 | HBV |
|  |  |  | plasma | microarray-qRT-PCR | 196 | 72 | 53±12 | HBV |
|  |  |  | plasma | microarray-qRT-PCR | 196 | 56 | 53±12 | HBV |
| Xu et al. [50] | 2011 | China | serum | qRT-PCR | 101 | 89 | 50 | 75%HBV |
|  |  |  | serum | qRT-PCR | 101 | 89 | 50 | 75%HBV |
|  |  |  | serum | qRT-PCR | 101 | 89 | 50 | 75%HBV |
| Qu et al. [51] | 2011 | USA | serum | qRT-PCR | 105 | 107 | 55 | 62.9%HCV |
|  |  |  | serum | qRT-PCR | 105 | 107 | 55 | 62.9%HCV |
| Li et al. [19] | 2010 | China | serum | sequencing-qRT-PCR | 65 | 100 | 53.4±9.1 | HBV |
|  |  |  | serum | sequencing-qRT-PCR | 65 | 75 | 53.4±9.1 | HBV |
|  |  |  | serum | sequencing-qRT-PCR | 55 | 50 | 52.83±7.85 | HBV |
|  |  |  | serum | sequencing-qRT-PCR | 55 | 50 | 52.83±7.85 | HBV |

| **First Author** | **Control Type** | **Internal reference** | **MiRNA assay** | **Cut-off** | **Status** | **Sensitivity** | **Specificity** |
| --- | --- | --- | --- | --- | --- | --- | --- |
| Ghosh et al.[25] | Chronic hepatitis | cel-miR-39 | miR-126 | 7.38 | up | 0.63 | 0.58 |
|  | Chronic hepatitis | cel-miR-39 | miR-142-3p | 7.45 | up | 0.32 | 0.91 |
| Amr et al.[49] | Chronic hepatitis | RNU48 | miR-21 | 3.8 | up | 1 | 0.812 |
|  | Chronic hepatitis | RNU48 | miR-199a | 0.685 | down | 0.545 | 1 |
| Hung et al.[48] | Dysplastic nodule | SnRNAU6 | miR122 | 0.4 | up | 0.667 | 0.567 |
|  | Dysplastic nodule | SnRNAU6 | let-7b | 3.6 | up | 0.825 | 0.467 |
|  | Dysplastic nodule | SnRNAU6 | let-7b miR122 | 0.66 | up | 0.848 | 0.5 |
| Chen et al.[47] | Chronic hepatitis | SnRNAU6 | miR-182 | 9.56 | up | 0.7864 | 0.9158 |
|  | Chronic hepatitis | SnRNAU6 | miR-331-3p | 5.55 | up | 0.7961 | 0.8947 |
| Lin et al.[46] | HBV Carriers | cel-miR-67 | 7 miRNAs ^a^ | NR | up | 0.857 | 0.833 |
|  | Chronic hepatitis | cel-miR-67 | 7 miRNAs ^a^ | NR | up | 0.745 | 0.899 |
| Yang et al.[45] | diseases/healthy | SnRNAU6 | miR218 | 0.61 | down | 0.667 | 0.691 |
| Zhuang et al.[44] | Chronic hepatitis | SnRNAU6 | miR-26a | 17 | down | 0.75 | 0.7 |
|  | Chronic hepatitis | SnRNAU6 | miR-101 | 10.67 | down | 0.549 | 0.769 |
|  | Chronic hepatitis | SnRNAU6 | miR-26a miR-101 | NG | down | 0.706 | 0.802 |
|  | Healthy | SnRNAU6 | miR-21 | 19.18 | up | 0.674 | 0.558 |
|  | Healthy | SnRNAU6 | miR-26a | 13.85 | down | 0.519 | 0.952 |
|  | Healthy | SnRNAU6 | miR-101 | 10.1 | down | 0.471 | 0.81 |
| Chen et al.[43] | cirrhosis | has-miR-16 | miR-338-5p | 1.743 | up | 0.745 | 0.828 |
|  | Healthy | has-miR-16 | miR-338-5p | 1.784 | up | 0.723 | 0.9968 |
|  | cirrhosis | has-miR-16 | miR-15b-5p | 1.007 | up | 0.681 | 0.79 |
|  | Healthy | has-miR-16 | miR-15b-5p | 0.807 | up | 0.872 | 0.742 |
|  | cirrhosis | has-miR-16 | miR-764 | 1.181 | up | 0.745 | 0.759 |
|  | Healthy | has-miR-16 | miR-764 | 1.465 | up | 0.702 | 0.839 |
| Yin et al.[42] | Healthy | SnRNAU6 | miR-375 | 1.192 | down | 0.523 | 0.727 |
|  | Healthy | SnRNAU6 | miR-199a-3p | 2.133 | down | 0.718 | 0.861 |
| Wen et al.[41] | HBV Carriers | cel-miR-39 | 8 miRNAs ^b^ | NR | up | 0.866 | 0.646 |
| Zhang et al.[40] | Healthy | SnRNAU6 | miR143 | 2.21 | up | 0.73 | 0.83 |
|  | Healthy | SnRNAU6 | miR215 | 4.62 | up | 0.8 | 0.91 |
| Li et al.[39] | Chronic hepatitis | has-miR-16 | MiRNA-139 | -3.24 | down | 0.806 | 0.581 |
| Tan et al.[38] | Chronic hepatitis | miR-24 | 8 miRNAs ^c^ | NR | more up | 0.816 | 0.846 |
|  | Healthy | miR-24 | 8 miRNAs ^c^ | NR | more up | 0.828 | 0.833 |
| Luo et al.[37] | HBV Carriers | SnRNAU6 | MiRNA-122a | 1.205 | down | 0.706 | 0.671 |
| Shen et al.[36] | 80%healthy | SnRNAU6 | miR-483-5p | NR | up | 0.551 | 0.857 |
|  | 80%healthy | SnRNAU6 | miR-483-5p/HCV | NR | up | 0.755 | 0.898 |
| Zhang et al.[35] | Chronic hepatitis | miR-156a | miR-483-5p | 2.824 | up | 0.7376 | 0.6607 |
|  | Chronic hepatitis | miR-156a | miR-500a | 1.83 | up | 0.74 | 0.51 |
| Tomimaru[3] | Chronic hepatitis | miR-16 | miR-21 | 0.754 | up | 0.611 | 0.833 |
|  | Healthy | miR-16 | miR-21 | -0.10 | up | 0.873 | 0.92 |
| Li et al.[34] | Chronic hepatitis | SnRNAU6 | miR-18a | 1.796 | up | 0.772 | 0.7 |
|  | Healthy | SnRNAU6 | miR-18a | 1.765 | up | 0.86 | 0.75 |
| Liu et al.[26] | HBV/healthy | NR | miR-15b | NR | up | 0.983 | 0.153 |
|  | HBV/healthy | NR | miR-130b | NR | up | 0.877 | 0.814 |
|  | HBV/healthy | NR | miR-15bmiR-130 | -0.61 | up | 0.983 | 0.915 |
| Qi et al.[21] | Healthy | miR-16 | miR-122 | 0.475 | up | 0.816 | 0.833 |
|  | Chronic hepatitis | miR-16 | miR-122 | 0.651 | up | 0.776 | 0.578 |
| Zhou et al.[5] | Healthy | NR | 7 miRNAs ^d^ | NR | more up | 0.832 | 0.939 |
|  | Chronic hepatitis | NR | 7 miRNAs ^d^ | NR | more up | 0.791 | 0.764 |
|  | Cirrhosis | NR | 7 miRNAs ^d^ | NR | more up | 0.75 | 0.911 |
| Xu et al.[50] | Healthy | NR | miR-21 | 0.46 | up | 0.84 | 0.735 |
|  | Healthy | NR | miR-122 | 0.7 | up | 0.707 | 0.691 |
|  | Healthy | NR | miR-223 | 1.91 | up | 0.8 | 0.765 |
| Qu et al.[51] | Chronic liver | SnRNAU6 | miR-16 | 6 | down | 0.721 | 0.888 |
|  | Chronic liver | SnRNAU6 | miR-199a | 10 | down | 0.629 | 0.935 |
| Li et al.[19] | Healthy | NR | 5miRNAs**^e^** | NR | up | 0.969 | 0.994 |
|  | HBV | NR | miR-10a,miR-125 | NR | up | 0.985 | 0.985 |
|  | cancer-free controls | NR | 3 miRNAs **^f^** | NR | up | 0.979 | 0.991 |
|  | cancer-free controls | NR | miR-375 | NR | up | 0.96 | 1 |

*SD* standard deviation, *NR* not reported, HBV hepatitis B virus, HCV hepatitis C virus **^a^** miR-29a miR-29c miR-133a miR143 miR-145 miR-192 miR-505

**^b^** miR-20a-5p miR-25-3p miR-30a-5p miR-92a-3p miR-132-3p miR-185-5p miR-320a miR-324-3p

**^c^** has-miR-206 has-miR-141-3p has-miR-433-3p has-miR-1228-5p has-miR-199a-5p has-miR-122-5p has-miR-192-5p has-miR-26a-5p

^d^ miR-122, miR-192, miR-21, miR-223, miR-26a, miR-27a and miR-801

^e^ miR-23b,miR-423, miR-375, miR-23a, and miR-342-3p

^f^ miR-375,miR-25,and let-7f
